# Maternal Bonding as a Protective Factor for Orthorexia Nervosa Risk in Dietetics Students

**DOI:** 10.3390/nu15163577

**Published:** 2023-08-14

**Authors:** Dafni Athanasaki, John Lakoumentas, Gregorio Paolo Milani, Carlo Agostoni, Florian Berghea, Marcela Daniela Ionescu, Emilia Vassilopoulou

**Affiliations:** 1 Department of Nutritional Sciences and Dietetics, International Hellenic University, 57400 Thessaloniki, Greece; daphnieath1999@gmail.com (D.A.); johnlakoo@gmail.com (J.L.); vassilopoulouemilia@gmail.com (E.V.); 2 Pediatric Unit, Fondazione IRCCS Ca’ Granda Ospedale Maggiore Policlinico, 20122 Milan, Italy; milani.gregoriop@unimi.it (G.P.M.); carlo.agostoni@unimi.it (C.A.); 3 Department of Clinical Sciences and Community Health, Università degli Studi di Milano, 20122 Milan, Italy; 4 Department of Internal Medicine and Rheumatology, Carol Davila University of Medicine and Pharmacy, 050474 Bucharest, Romania; drmarcela.ionescu@gmail.com; 5 Spital Clinic Sf. Maria, Bd. Ion Mihalache 37-39, Et 1 Secretariat, 011172 Bucharest, Romania; 6 “Marie S. Curie” Emergency Children’s Clinical Hospital, 041451 Bucharest, Romania

**Keywords:** orthorexia nervosa, eating disorders, body image, anxiety, body mass index, parental bonding relationship, exercise, dietetics students

## Abstract

This study aimed to determine the prevalence of the risk of orthorexia nervosa (ON) in dietetics students in Greece, and its relationship with diet, risk of eating disorder (ED), body mass index (BMI), body image flexibility, and parental attachment. The participants were 132 dietetics students, with a mean age of 22.94 ± 3.5 years, who completed a series of questionnaires that recorded sociodemographic, clinical, and anthropometric characteristics; adherence to the Mediterranean diet (MedDiet); ON indicators as determined by the ORTO-15 questionnaire; body image flexibility, with the Body Image-Acceptance and Action Questionnaire (BI-AAQ-5); the risk for ED as assessed using the EAT-26; anxiety level according to the STAIT 6 instrument; and recollection of their parents’ attitudes towards them during the first 16 years of life, with the Parental Bonding Instrument (PBI). ON risk appeared to be significantly associated with BMI (*p* = 0.002), exercise frequency (*p* = 0.023), anxiety level (*p* = 0.002), risk of ED (*p* < 0.001), body image inflexibility (*p* < 0.001), and inversely with the affectionate constraint of maternal bonding (*p* = 0.036). In conclusion, disordered eating behaviors and body shape concerns are prevalent among dietetics students, with parental attachment to the mother influencing their occurrence. Identification of potential ON and development of prevention mechanisms during childhood could help eliminate these concerns and improve the lives of dietetics students.

## 1. Introduction

Self-imposed dietary rules intended to promote health, as reported in 1997 by Bratman, may, in some cases, conversely have detrimental effects [1]. The pursuit of “extreme dietary purity”, with an exaggerated focus on food, may lead to specific disordered eating (DE) behavior, which Bratman called orthorexia nervosa (ON). Orthorexia is a neologism derived from the Greek words *orthos* (correct) and *orexis* (appetite), with the literal meaning “correct appetite for food”. The expression was coined to indicate a possible new eating disorder (ED), the core symptom of which is a dangerous, excessive, obsessive focus on eating foods perceived as healthy [2]. The key features of ON that have been proposed include: (a) an obsessive focus on eating practices believed to promote optimal well-being through healthy eating, with inflexible eating rules, recurrent and persistent food-related preoccupation, and compulsive behavior; (b) subsequent clinically significant impairment (e.g., medical or psychological complications, great distress, and/or defects in important areas of functioning) [2,3]. An excessive focus on food quality, food preparation [4], agricultural methods, and the data on food labels is another characteristic feature of ON [3]. This attitude leads to impairment of social behavior and social relationships and can have an impact on the physical status and activities of the individual. ON must be differentiated from healthy orthorexia, which is defined as a “healthy interest in diet, healthy behavior with regard to diet, and eating healthily as part of one’s identity” [5]. ON has not yet been included as a diagnostic category in the Diagnostic and Statistical Manual of Mental Disorders (DSM-5), or the International Classification of Diseases-0 [5]. Perfectionism, obsessive-compulsive traits, other psychopathology, DE, the history of a diagnosed ED, dieting, poor body image, and a drive for thinness have all been positively associated with a greater likelihood of ON [6].

EDs are defined as disruptions in eating behavior, with excessive concern about body weight to a degree that impairs physical health and/or psychosocial functioning. EDs can present as severe psychiatric illnesses, associated with high rates of morbidity and mortality [7], with anorexia nervosa (AN) showing the highest mortality rate of any psychiatric disease [8].

Several different forms of ED behaviors may be followed by individuals who do not meet the criteria for a particular ED, such as binge eating, food restraint, emotional eating, disinhibition, strict dieting, and control of body weight and shape, through inappropriate compensatory behavior [9,10]. Unhealthy eating practices, such as extreme dieting, fasting, induced vomiting, and abuse of laxatives, are considered risk factors [10], although in isolation, they do not warrant the psychiatric diagnosis of an ED [9].

Studies worldwide have reported a high prevalence of EDs in both dietetics students and dietitians [8,10,11,12,13,14], and both populations have been characterized as having a high risk of DE [11,15] and an obsession with healthy eating behavior [16], and the development of ON [8,17]. In a study comparing eating behavior in Portuguese undergraduate nutrition students and students taking other courses, nutrition students showed higher levels of dietary restraint and binge eating than students in other courses [18].

Regarding the prevalence of ON, high rates have been reported among dietetics students and dietitians [8]. Orthorexic tendencies [19] and the development of orthorexic behavior [20] are reported to be more strongly represented in people in nutrition studies than in the general population [16], with a prevalence varying from 6.9% in the general population to over 80.0% in certain groups, such as dietitians and dietetics students [19].

In certain other fields, a high prevalence of ON has been reported, reaching close to 90.0% among high-risk populations, such as ballet dancers and athletes. It is even observed in health workers [21], and Fidan and colleagues [22] report a high prevalence of orthorexia in medical students (43.6%).

Other studies, however, have reported different findings. In a study of 189 female Portuguese students aged 18–25 years, no difference in the risk of developing ED was demonstrated between students majoring in nutrition and other health-related or non-health-related majors [23]. Some researchers argue that an increase in nutritional knowledge is related to a healthier diet and better food choices and, therefore, conclude that individuals trained in nutrition and dietetics are more likely to develop healthy eating behavior [8] and may be at a lower risk of developing DE behavior than non-nutrition majors [10]. The high prevalence of unhealthy behavior reported in some studies of dieticians and dietetics students in their relationship with food, however, indicates a need for further investigation of factors that may influence this group in the development of DE behavior.

Parents play a crucial role in helping both adolescents and young adults to master the tasks associated with their development to maturity [24], and several characteristics of adverse parenting styles have been associated with DE in the offspring [25]. The findings of several studies suggest that a particular parenting style that is warm, involved, and characterized by disciplinary methods that are supportive rather than punitive, and is firm and consistent in setting and enforcing guidelines and boundaries, with developmentally appropriate expectations, is advantageous in supporting adolescents. It has been suggested that one stereotypical parenting style during the first 16 years, characterized by low caring and high control, was a common feature of patients with AN [26] and an association has been documented between low levels of parental care and psychopathological symptoms, weight phobia, and poor body image [24]. Anxiety may be a feature of DE, and studies have shown that DE attitudes are significantly correlated with anxiety levels [27] and that levels of anxiety are higher in subjects at high risk of ED [28,29].

This study aimed to explore factors that may lead to ON risk in dietetics students in Greece and their risk of developing ED, including disturbed eating patterns, levels of anxiety, and their parental bonding relationships.

## 2. Materials and Methods

### 2.1. Participants and Procedures

An observational study was conducted with students of the Department of Nutritional Sciences and Dietetics at the International Hellenic University (IHU). The participants were students from all four years of the study course, and some who had exceeded the statutory years of study. All the students were fully informed of the scope and procedures of the study, and those who wished to participate provided written informed consent. Of the total sample of 497 participants, 132 entered the study after exclusion. Exclusion of participants from the original sample was due to either lack of consent or the presence of a chronic disease that required a specialized diet. The study was conducted with the permission of the Committee for Research Ethics of the Aristotle University of Thessaloniki (1.272/20.10.2020) and in accordance with the Code of Ethics of the World Medical Association (Declaration of Helsinki).

### 2.2. Sociodemographic, Medical, and Anthropometric Characteristics

Self-reported information was collected from each participant through face-to-face interviews conducted by members of the research team. The data collected comprised (a) anthropometric characteristics, including age, body weight, and height of the participant and the participant’s mother and father, based on which the respective body mass index (BMI) was calculated as Kg/m^2^, and categorized according to the WHO categorization (using in our study the three resulting categories: <18.5: underweight; 18.5–24.9: normal weight; >25.0 overweight, denoting pre-obesity or obesity); [30] (b) demographic information, including years of schooling, and the frequency, duration, and level of physical activity; (c) medical history, including chronic illness and medication. Specific instruments were administered to measure diet, anxiety level, body image, parental attachment, risk of eating disorder, ON, circadian rhythm, and mood processes, as listed below.

### 2.3. Study Instruments

#### 2.3.1. Mediterranean Diet (MedDiet) Score

Dietary habits were explored by assessing compliance with the Mediterranean diet (MedDiet). The MedDiet scoring system, which was developed by Panagiotakos and colleagues [31] in Greece, includes the following 11 food groups: unrefined cereals (wholemeal bread, pasta, rice, other cereals, biscuits, etc.), fruits, vegetables, legumes, potatoes, fish, meat and meat products, poultry, full-fat dairy products (such as cheese, yogurt, and milk), olive oil, and alcohol. A score of between 0 and 5 is assigned to each item in most of the 11 food groups according to the frequency of consumption per month (i.e., never, 1–4, 5–8, 9–12, 13–18 and >18 servings per month are scored 0, 1, 2, 3, 4, and 5, respectively) [32]. For some of the items, specifically, red meat and meat products, poultry, and full-fat dairy products, a score of 0–5 is assigned for reported consumption using a reverse scale. A score of 0–5 is assigned for olive oil use, both in cooking and meal preparation, corresponding to “never”, “rarely”, “<once/week”, “1–3 times/week”, “3–5 times/week”, and “daily”, respectively. For alcohol (all alcoholic beverages), a score of 5 is given for “no consumption” or “consumption of <300 mL/day”, 0 for “consumption >700 mL/day”, and scores of 4 to 1 for consumption of “300–400 mL”, “400–500 mL”, “500–600 mL”, and “600–700 mL/day”, respectively [31,33]. The total MedDiet score ranges from 0 to 55, with higher values indicating closer adherence to the MedDiet [32]. For the purposes of this study, adherence to the MedDiet was divided into two levels: insufficient (0–27) and sufficient (28–55) [31,33].

#### 2.3.2. ORTO-15 Questionnaire

The Greek version of ORTO-15 [34], a self-reported questionnaire developed by Donini and colleagues [35] consisting of 15 multiple-choice questions, six of which are from the Bratman scale [36], was used to measure the prevalence of orthorexia. Responses were scored on a 4-point Likert-type scale, where ‘always’ = 1, ‘often’ = 2, ‘sometimes’ = 3, and ‘never’ = 4. According to the authors’ original instructions, items 2, 5, 8, and 9 are scored inversely (i.e., ‘always’ = 4, ‘often’ = 3, ‘sometimes’ = 2, ‘never’ = 1). Items 1 and 13 are scored as follows: “always” = 2, “often” = 4, “sometimes” = 3, and “never” = 1. A lower score indicates a higher level of ON symptomatology [34]. Scores above 40 indicate the absence of ON. The authors suggest a threshold of 40 for the diagnosis of the risk of ON. The reliability analysis of the ORTO-15 questionnaire in the current population provided a reliable Cronbach α equal to 0.65 (95% CI: 0.56–0.73).

#### 2.3.3. Eating Attitudes Test (EAT-26)

The Greek version of the self-reported Eating Attitudes Test (EAT-26) was used [37] to measure symptoms and concerns about ED. It consists of a 26-item scale, divided in three sections: (a) self-reported height and weight, used to calculate BMI; (b) 26 items related to how often the respondent engages in certain behaviors, scored on a 6-point Likert scale (“always”, “usually”, “often”, “sometimes”, “rarely”, and “never”); and (c) 5 behavioral items on a 6-point Likert scale, to explore how often the respondent has engaged in disordered eating behaviors in the past 6 months (‘never’, ‘once a month or less’, ‘2–3 times a month’, ‘once a week’, ‘2–6 times a week’, and ‘once a day or more’). Only the last two sections of the questionnaire were used in this study, as height and weight were measured by the researchers. For this study, the responses for items 1–25 were scored on a 4-point scale with “always” receiving three points, “usually” receiving two points, “often” receiving one point, and “sometimes,” “rarely,” and “never” receiving zero points. Item 26 is scored in reverse, and the final score is calculated as the sum of items 1–26. A score of 20 or higher on the EAT-26 reflects a high level of concern about diet, body weight, or problematic eating behavior [38].

#### 2.3.4. The Body Image-Acceptance and Action Questionnaire 5 (BI-AAQ-5)

The Greek version of the Body Image-Acceptance and Action Questionnaire 5 (BI-AAQ-5) was used to assess body image flexibility, which is associated with greater psychological flexibility, reduced body image dissatisfaction, and less DE [39]. The BI-AAQ was customized from pre-existing psychological flexibility measures, specifically to evaluate responsiveness to body-related thoughts and emotions [40]. The BI-AAQ-5 [41] is a shortened version of the original instrument and includes five items scored on a scale ranging from 1 (never true) to 7 (always true) [42]. The items are negatively worded, so reverse scoring of each item is required before all items are summed to determine the total score. The short form has been shown to perform as well as the long form in terms of its factorial structure and its association with theoretically relevant constructs, including body image dissatisfaction, stigma, internalization of social norms of appearance, self-compassion, and poor mental health. Scores of 19 or above indicate a potential risk of distorted body image [41]. The participants in this study also filled out a questionnaire about their perceptions of their mother’s and father’s flexibility of body image.

#### 2.3.5. STAI

The Greek version of the STAI questionnaire [43] was used to measure two different concepts of anxiety: situational anxiety (STAI-S) and trait anxiety (STAI-T). The STAI-T scale consists of 20 statements describing how respondents feel in general, and it is the one used in the research. The STAI’s validity requires that the examinee have a clear understanding of the “state” and “trait” instructions. Each question is scored on a 4-point scale (“not at all”, “a little”, “moderately”, “very much”). The range of scores for Form Y of the STAI ranges from a minimum score of 20 to a maximum score of 80. STAI scores are typically classified as “no or low anxiety” (20–37), “moderate anxiety” (38–44), and “high anxiety” (45–80) [44]. The STAI-S has been proven to be a valid and reliable measure of situational anxiety [45]. Our study used the six-item short form of the STAI (STAI-6), which produces results similar to those obtained using the full form, with reliability and validity [46].

#### 2.3.6. The Parental Bonding Instrument (PBI)

The Greek version of the Parental Bonding Instrument (PBI) was used to retrospectively assess how participants remembered their parents during the first 16 years of their lives [47,48]. Its two constituent scales measure “caring” and “overprotection and control” as fundamental parenting styles, as experienced by the child, and were designed to be filled in separately by mothers and fathers. It consists of 25 items, including 12 caring items and 13 overprotection items, classified into high or low categories according to the following cut-off scores: for mothers, a caring score of 27.0 and an overprotection score of 13.5; for fathers, a caring score of 24.0 and an overprotection score of 12.5. These cut-off scores categorize parenting styles into non-affectionate control (high control–low care), optimal (low control–high care), affective restraint (high care–high control), and neglectful parenting (low care–low control) [24]. The PBI was completed separately for each parent.

#### 2.3.7. Statistical Analysis

The mixed dataset from the set of questionnaires comprised both quantitative and qualitative variables. On application of the Shapiro–Wilk test for composite normality, the quantitative variables were all found to follow a normal distribution and were expressed as mean ± standard deviation (SD). The qualitative variables were expressed as absolute count (percent %). To explore the association of ON risk with potential predictive factors, Student’s *t*-test was applied in the case of quantitative factors, and Pearson’s chi-squared test of independence in the case of qualitative factors. Multivariate binomial logistic regression analysis was applied, following imputation of a small fraction of missing values via the random forest method [49], to assess the combined effect of the predictors on the risk of ON. Statistical significance was set at *p* < 0.05. All statistical processes were implemented with R (4.1.2 2021-11-01) and the RStudio IDE (2021.09.1+372), both of which are open-source software products for data analysis.

## 3. Results

### 3.1. Characteristics of the Study Population

Among the 132 respondents (26.5%) whose data were analyzed, 120 were female (90.9%) and 12 were male (9.1%), and their mean age was 22.94 ± 3.5 years.

According to their BMI, 17 were underweight (13.0%), 93 were normal weight (71.0%), and 21 were overweight (16.0%).

Of the 132 participants, 109 (82.6%) had completed more than two years of study, and 23 (17.4%) had completed less than two years.

Concerning disease and medication, 123 (93.2%) participants reported that they had no chronic disease, and 113 (88.3%) reported that they were taking no medication.

Regarding physical activity, 110 (85.3%) reported exercising four or fewer days per week, with the majority reporting sessions of over one hour (n = 81; 63.8%).

According to the parents’ BMI as reported by the students, 55 mothers (47.8%) were classified as normal weight and 60 (52.2%) as overweight, and 24 (20.9%) fathers were normal weight and 91 (79.1%) overweight.

According to their MedDiet scores, 26 (19.7%) participants showed insufficient adherence, while the remaining 106 (80.3%) showed sufficient adherence.

### 3.2. Risk of ON, ED, and Body Image Dissatisfaction, Stress Level, and Parental Bonding Relationship

The mean score of the 132 participants on the ORTO-15 questionnaire was 38.16 ± 4.99, and 74 (56.1%) of the participants presented symptoms of ON, with scores of ≤40. According to their responses on the EAT-26 questionnaire, a high level of concern was registered by 30 (22.7%) participants, and according to their scores on the BI-AAQ-5, 34 (27.0%) expressed body image dissatisfaction.

Only 48 (37.2%) participants reported no or low anxiety, with the rest recording moderate (n = 32; 24.8%) and high levels of anxiety (n = 49; 38.0%) according to the STAIT questionnaire.

In terms of parental relationships, the PBI revealed that optimal parenting with the mother was recalled by 68 (53.6%) participants, neglectful parenting by 20 (15.7%), affectionless control by 28 (22.0%), and affectionate constraint by 11 (8.7%). The perceived risk of body image inflexibility in the mother, assessed using the BI-AAQ-5, completed by the child, was high in 73 (55.3%) participants.

The corresponding PBI scores of the fathers revealed that optimal parenting was recalled in 54 (43.2%) participants, neglectful parenting in 24 (19.2%), affectionless control in 33 (26.4%), and affectionate constraint in 14 (11.2%). The perceived risk of body image inflexibility in the father, according to the child’s responses to the BI-AAQ-5 questionnaire, was high in 106 (80.3%) participants.

### 3.3. Factors That Influence the Presence of the Risk of ON

To identify the factors associated with the risk of ON, we divided the study sample into two groups based on the presence or absence of ON risk, according to a cut-off score of 40 on their responses to the ORTO-15 questionnaire.

Table 1 presents the factors associated with the risk of ON, as assessed by the ORTO-15 questionnaire, according to bivariate analysis. The presence of ON risk was statistically significantly associated with BMI, frequency of exercise, anxiety level, risk of ED, and body image inflexibility.

In the orthorexic group, 3 participants (4.1%) were underweight, 55 (75.4%) were of normal weight, and 15 (20.5%) were overweight, with a mean BMI of 22.87 ± 3.27. The risk of ON was strongly correlated with BMI. Specifically, participants who were normal and overweight scored higher for the risk of ON (*p* = 0.002).

Exercise taken more than 4 times per week was associated with the presence of the risk of ON (*p* = 0.023), with 15/74 orthorexic participants (21.1%) exercising at this frequency.

ON appeared to be influenced by anxiety, according to the STAIT score (*p* = 0.019). Of the ON risk group, only 19/74 (26.7%) recorded no anxiety, or a low level of anxiety, while 19/74 (26.7%) recorded moderate and 33/74 (46.6%) high levels.

The prevalence of risk of ED according to EAT-26 was 36.5% (n = 27) in the orthorexic group compared with 5.2% (n = 3) in the non-orthorexic group, with mean scores of 19.85 ± 9.62 and 12.64 ± 4.99, respectively. Symptoms and concerns about ED, according to EAT-26, were strongly associated with ON risk (*p* < 0.001).

A percentage of 42.3% (n = 30) of the participants with ON risk and only 7.3% (n = 4) of those without ON risk showed body image inflexibility. A higher degree of body image inflexibility was associated with an increase in the symptoms of ON (*p* < 0.001).

### 3.4. The Association of the Risk of ON with BMI, Body Image Inflexibility, Risk of ED, Anxiety Level, and Maternal Bonding Relationship

**Figure 1:**
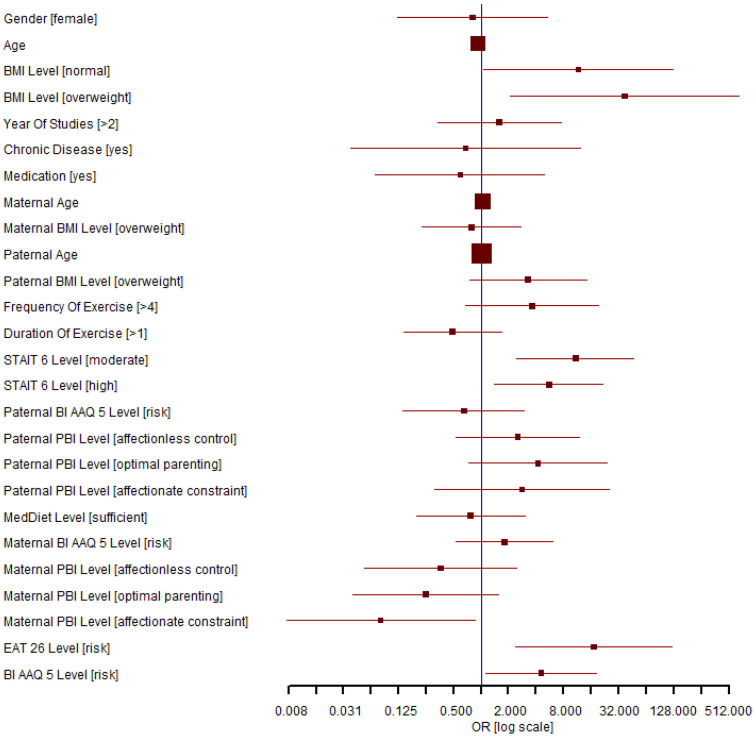
The forest plot that corresponds to the logistic regression model, linking the set of predictors with ON risk in Greek students of nutrition and dietetics, as assessed by the ORTO-15 questionnaire (N = 132). BMI = body mass index; STAIT-6 = State-Trait Anxiety Inventory; BI-AAQ-5 = Body Image-Acceptance and Action Questionnaire-5; PBI = Parental Bonding Instrument; MedDiet = Mediterranean diet; EAT-26 = Eating Attitudes Test-26.

All variables were entered into the multivariate logistic regression model. The results are presented in Table S1, and the logistic regression forest plot of the variables explored about ON is presented in Figure 1.

According to the results of multivariate analysis, ON risk was statistically significantly associated with BMI, anxiety level as measured by STAIT, risk of ED, and body image inflexibility as measured by BI-AAQ-5, and inversely with maternal bonding.

Regarding BMI, normal-weight and overweight students had a high incidence of ON risk, with *p* = 0.044 and *p* = 0.014, respectively.

Despite the results of the bivariate analysis, exercise frequency did not appear to be associated with ON risk in multivariate analysis.

Both the risk for body image inflexibility and ED were associated with ON risk; *p* = 0.034 for the BI-AAQ-5 score and *p* = 0.005 for the EAT-26 score.

The anxiety level as measured by STAIT strongly correlated with ON risk for both moderate and high levels of anxiety (*p* = 0.002 and *p* = 0.014, respectively).

A statistically significant correlation was revealed between the non-indication of ON risk and maternal bonding in the first 16 years of life, as evaluated using the PBI (Table S1). Maternal PBI questionnaire revealed that the three (non-base) levels of affectionless control, optimal parenting, and affectionate constraint favored the reduced risk of ON, but was statistically significant only for the affectionate constraint type (*p* = 0.036).

A statistically significant outcome is considered when the horizontal line of the predictor does not cross the vertical line corresponding to OR = 1. That is, either the red horizontal line is completely on the left of the vertical blue line, denoting no indication of ON risk (*p* < 0.05 and 95% CI of OR strictly smaller than 1), or the red horizontal line is completely on the right of the vertical blue line, denoting indication of ON risk (*p* < 0.05 and 95% CI of OR strictly bigger than 1).

## 4. Discussion

Students of nutrition and dietetics bear the multifactorial burden of acquiring nutrition and health information, conforming to the healthy ideals that they are learning, and providing a role model [50]. This experience [51] is a probable reason for the observation that ON and EDs are more common among dieticians and students of nutrition and dietetics than in the general population [8,17,19,52,53,54]. The present study on Greek students of nutrition and dietetics reported both a high incidence of risk for ON (56.1%) and a substantial risk for EDs (22.7%) in this population. The reduced risk of ON, though, was correlated herein with maternal bonding characterized by affectionate constraint.

Previous studies reported a high prevalence of ON ranging from 41.9% to 88.7% [8,16,17,20,51,55], and a prevalence of ED ranging from 12.9% to 15.4% among dietetics students and dieticians [12,17]. An international study with data from 14 countries found that 77.0% of dieticians believe that EDs are a problem for dietetics students [11], and a study in South Africa observed a higher risk of EDs in dietetics students than in students in other fields [14,56]. Kassier and Veldman showed a higher risk of ED among first-year nutrition and dietetics students (33.3%) in South Africa compared with students in non-nutrition-related courses (16.9%) [14]. Dieticians face additional challenges related to having a professional function that involves evaluation of eating choices, and separation of professional knowledge from personal beliefs surrounding food [17].

The majority of our participants (71.0%) were of normal weight according to the WHO BMI classification, in line with other studies reporting similar rates for dietetics students [14,56] but in contrast with the lower rate (54.0%) reported by Rusil and Harith. Another study suggested that dietetics students are no more or less prone to negative attitudes towards obesity than their peers [57,58]. We identified a strong correlation in our sample between BMI and ON risk, with both normal-weight and overweight participants at risk of ON. This correlation is consistent with previous studies that identified BMI as a key factor influencing an individual’s orthorexic tendency [59]. Moreover, in several study samples, when BMI increases, ON increases, specifically in Turkish dieticians [16], college students and nutritionists [55], Greek dietetics students [51], and Turkish medical students [22]. The strongest correlation we found between increased BMI and orthorexia may be that a person who is overweight or obese may feel insecure with their body image, which drives them to eat only healthy foods [22]. Other studies, however, on undergraduate students [60,61] and registered dieticians [17], while reporting an association between BMI and ON, suggested that underweight participants were at a greater risk of ON. In contrast to these findings, Cinosi and colleagues [62] and Bağci Bosi and colleagues [63] found no association between BMI and ORTO-15 score in Italian adults and medical doctors in Turkey, respectively [64], and other studies have also reported that BMI appeared to not affect the presence of ON [6,64,65,66,67,68]. Although findings, to date, on how BMI is associated with ON are contradictory [6], our study revealed a correlation, and we conclude that the effect of BMI on food choice can be interpreted as a result of the efforts made to achieve ideal body weight [64].

The high incidence of EDs documented among nutrition and dietetics students and professionals has been suggested to be due in part to their frequent exposure to food and nutrition information [15,51]. In our sample of Greek dietetics students, the risk of ED was strongly associated with the presence of ON risk. As the EAT-40 score increased, the ORTO-15 score decreased, indicating an increased tendency towards an ED. This association is consistent with previous studies reporting lower ORTO-15 scores in groups of individuals with ED in studies on dieticians [6,8,16,17,19,64]. Caferoglu and Toklu [8] confirmed the association between the EAT-26 score and the tendency for ON and suggested that EDs could increase the risk of ON by five times. McComb and Mills [6] also suggested that participants with a current or previous ED were particularly likely to have ON, with prevalence rates ranging from 28.0–82.7% in clinical samples of Polish and Italian patients with an ED.

The percentage of participants in our study who exercised more than 4 times per week was 14.7%, a rate similar to that reported in dietetics students at USZA (19.0%) for high levels of physical activity [58]. In line with previous studies, high exercise frequency (>4 times/week) was associated with the presence of ON risk. Studies have documented that orthorexic behavior is associated with the level of physical activity, showing a positive correlation with the frequency and duration of exercise [69,70,71,72].

The orthorexic individuals in our study recorded moderate and high levels of anxiety. The strong correlation between ON and anxiety, measured according to the STAIT, is in agreement with previous studies linking high levels of anxiety with ON [2,73,74,75,76]. This may indicate that, on the basis of their eating behavior, a generalized state of anxiety prevails that is compensated for through food, rather than eating in response to a physiological stimulus or for pleasure [67]. ED can manifest as a result of dealing with stress, and it has been suggested that dietitians may experience ED due to the high stress associated with their position and their intense focus on weight and personal struggles with food and diets. This may indicate that anxiety as a mediating factor, in addition to nutrition knowledge, contributes to high rates of ED or patterns of ED among practicing dietitians [15].

Consistent with previous studies that have reported a 26.7% rate of body image dissatisfaction [56], body image-related obsessions [51], and dissatisfaction with their physical appearance [11] among students training in nutrition and dietetics, a prevalence of 27.0% was found regarding the risk of body image inflexibility in the Greek students of nutrition and dietetics in our study. Other studies have shown ON to be positively correlated with body image attitudes [77], body dysmorphia [6], and body shape preoccupation [78], and lower scores for body area satisfaction [79]. Among Spanish university students, body dissatisfaction was found to be more likely in those with ON than in those without ON [6,80]. Appearance evaluation was also found to be negatively correlated with ON tendencies in Spanish university students [6,81]. Among Portuguese fitness participants also, those with ON were more likely to be dissatisfied with their physical appearance than those without ON [6,82]. In contrast, feelings of attractiveness and body dissatisfaction did not predict ON symptoms in Polish patients with ED [6,83]. As weight is a modifiable aspect of general appearance, it becomes a target of criticism from both self and society [15].

In the present study, there was no significant difference between males and females on the ORTO-15 score, which is consistent with previous research that found no gender difference on the ORTO-15 among artists [68] or resident medical doctors [6,63]. No relationship was found between gender and ON in samples from the United States, Australia [79,84], Portugal [82], or Greece [51]. Other studies have reported that ON is affected by gender, with a higher prevalence of orthorexia for males [55], or, conversely, for females [22,64,79].

The vast majority of participants in our study (80.3%) reported adequate adherence to the MedDiet, in line with previous studies reporting rates of 58.4% for Spanish college students [85] and 59.0% for Lebanese students [86], for good and adequate adherence, respectively. Other studies, however, have reported low to adequate adherence among Greek university students [87] and Cypriot students [88]. Strahler and colleagues [73] and Martinovic and colleagues [89] reported that the MedDiet, which is characterized by the use of olive oil as a main source of fat, and eating plenty of vegetables, nuts, fish, and white meat instead of red meat, may be associated with ON, as it was reported that this diet was more common among people with ON than those without ON [6]. In our study, we found no association between ON and adherence to the MedDiet because there was no difference related to adherence to this dietary pattern between the two groups.

The prevalence of ON risk showed no difference according to years of study, as was found in other reports [51,90]; a higher prevalence of disordered eating was reported in first-year dietetics students [14], and improvement in unhealthy eating practices over the course of the semester’s [15].

In our study sample, when the mother’s parenting style, as perceived by the child, was an affectionate constraint, there was a reduced risk of ON. A previous study reported that high maternal control is associated with higher odds of depressive, anxiety, eating, and behavioral disorders [91]. However, our finding may be explained with the description of Kong and Yasmin [92], mentioning that authoritarian parents use strict rules and constraints shaped by an excessive level of authority to control their children’s behavior. Thus, children follow the rules without questioning them, and their behavior is basically regulated by their parents [92]. Parental influences emerged in a previous study as being significant in the development of ON tendencies [93]. There are several reasons for this, including what two researchers described as “nurturing issues”, from which they concluded that parents were emotionally or physically unavailable and that a challenging relationship with parents could be expressed through controlled eating behavior [93]. The most frequent parenting style reported by patients with ED is low care and high control, and between 8.6% and 12.9% of these patients perceive their parents’ style as neglectful [24]. Patients who perceived neglectful maternal parenting during their first 16 years recorded higher scores in the subscales of drive for thinness and body dissatisfaction than those who perceived affectionless control and, in contrast to our findings, affectionate constraint styles [24].

In the present study, we investigated possible factors with a potential impact on the incidence of ON risk in Greek dietetics students studying exclusively in the Department of Nutrition Science and Dietetics at the IHU. The strength of our study lies in our attempt to link ON risk with a variety of factors related to both parental and study-related factors, risk of ED, and body image perception, which resulted in the identification of the significance of parental relationship on the development or protection of ON and opens a new ground for further research. In addition, healthy eating behavior should be maintained to improve quality of life, without being allowed to become an obsession [16]. Nutrition education could be expected to potentially improve an individual’s eating behavior, but the obsession with healthy eating may eventually lead to a decline in general health, with negative psychological and physiological consequences.

This study has some limitations. One limitation is that the questionnaires were self-reported, and the participants answered for themselves and their parents, increasing the likelihood of bias. A significant limitation of our work is the use of the ORTO-15 to assess ON. ORTO-15 has received criticism about its reliability and validity, and other measures are instead suggested [35,94]. Nevertheless, it is still the most widely used questionnaire for studies with ON. Additionally, the assessment of body fat was limited to the calculation of BMI, which may not be fully informative because it does not take muscle mass into account. Different methods of measuring it, such as the participants’ skinfolds, waist circumference, or waist-to-hip ratio, would be interesting to study. An additional limitation is that we did not explore the effect of the use of social media [95] or dating apps [96], presented as significant risk factors for ON by other investigators.

## 5. Conclusions

The results of the present study showed a considerable risk of ON and eating disorders in a sample of university students in nutrition and dietetics. The association of ON risk with BMI, exercise frequency, stress level, risk of ED, risk of body image inflexibility, and type of attachment with the mother indicates the wide spectrum of factors involved in this disorder. Further studies are needed to determine the causes of the increasing prevalence of ON and EDs in dieticians and students of nutrition and dietetics. Protective strategies are recommended, such as providing targeted nutrition education, and monitoring by experts of the health and nutrition messages these young people are receiving during their studies.

## Figures and Tables

**Table 1:** Characteristics of Greek students of nutrition and dietetics (N = 132), according to classification into orthorexia nervosa (ON) risk and non-orthorexia nervosa (non-ON) risk, based on scores on the ORTO-15 questionnaire.

Variable	Non-ON (N = 58)	ON (N = 74)	*p* Value
Gender			0.166
Male	3 (5.2%)	9 (12.2%)	
Female	55 (94.8%)	65 (87.8%)	
Age (years)	23.29 ± 4.13	22.66 ± 2.92	0.304
BMI (kg/m^2^)			0.002 *
Underweight	14 (24.1%)	3 (4.1%)	
Normal weight	38 (65.6%)	55 (75.4%)	
Overweight	6 (10.3%)	15 (20.5%)	
Years of Study			0.680
≤2	11 (19.0%)	12 (16.2%)	
>2	47 (81.0%)	62 (83.8%)	
Chronic Disease			0.155
No	52 (89.7%)	71 (95.9%)	
Yes	6 (10.3%)	3 (4.1%)	
Medication			0.180
No	47 (83.9%)	66 (91.7%)	
Yes	9 (16.1%)	6 (8.3%)	
Frequency of Exercise (sessions/week)			0.023 *
≤4	54 (93.1%)	56 (78.9%)	
>4	4 (6.9%)	15 (21.1%)	
Duration of Exercise (hours/session)			0.474
≤1	18 (32.7%)	28 (38.9%)	
>1	37 (67.3%)	44 (61.1%)	
Anxiety Level (STAIT)			0.019 *
None or low	29 (50.0%)	19 (26.7%)	
Moderate	13 (22.4%)	19 (26.7%)	
High	16 (27.6%)	33 (46.6%)	
MedDiet adherence			0.131
Insufficient	8 (13.8%)	18 (24.3%)	
Sufficient	50 (86.2%)	56 (75.7%)	
Eating Disorders concerns (EAT-26)			<0.001 *
No risk	55 (94.8%)	47 (63.5%)	
Risk	3 (5.2%)	27 (36.5%)	
Body image inflexibility (BI-AAQ-5)			<0.001 *
No risk	51 (92.7%)	41 (57.7%)	
Risk	4 (7.3%)	30 (42.3%)	
Maternal Age (years)	51.72 ± 5.62	51.71 ± 5.17	0.991
Maternal BMI (kg/m^2^)			0.527
Normal weight	25 (49.0%)	30 (46.9%)	
Overweight	26 (51.0%)	34 (53.1%)	
Maternal body image inflexibility (BI-AAQ-5)			0.704
No risk	27 (46.6%)	32 (43.2%)	
Risk	31 (53.4%)	42 (56.8%)	
Maternal bonding (PBI)			0.887
Neglectful parenting	8 (14.3%)	12 (16.9%)	
Affectionless control	12 (21.4%)	16 (22.5%)	
Optimal parenting	30 (53.6%)	38 (53.6%)	
Affectionate constraint	6 (10.7%)	5 (7.0%)	
Paternal Age (years)	55.83 ± 6.99	56.18 ± 6.23	0.773
Paternal BMI (kg/m^2^)			0.099
Normal weight	14 (28.0%)	10 (15.4%)	
Overweight	36 (72.0%)	55 (84.6%)	
Paternal body image inflexibility (BI-AAQ-5)			0.285
No risk	9 (15.5%)	17 (23.0%)	
Risk	49 (84.5%)	57 (77.0%)	
Paternal bonding (PBI)			0.244
Neglectful parenting	12 (21.4%)	12 (17.4%)	
Affectionless control	11 (19.6%)	22 (31.9%)	
Optimal parenting	24 (42.9%)	30 (43.5%)	
Affectionate constraint	9 (16.1%)	5 (7.2%)	

STAIT = State-Trait Anxiety Inventory; MedDiet = Mediterranean diet; EAT-26 = Eating Attitudes Test-26; BI-AAQ-5 = Body Image-Acceptance and Action Questionnaire-5; PBI = Parental Bonding Instrument; BMI = body mass index. * *p* ≤ 0.05 was considered statistically significant.

## Data Availability

Data are available upon request from the corresponding author.

## Supplementary Materials

The following supporting information can be downloaded at: https://www.mdpi.com/article/10.3390/nu15163577/s1, Table S1: Association of ON risk as assessed by the ORTO-15 in Greek students of nutrition and dietetics (N = 132) simultaneously with multiple variables (multivariate logistic regression analysis).
