# Supplementary material for: Maternal Bonding as a Protective Factor for Orthorexia Nervosa Risk in Dietetics Students
Source: Nutrients. 2023 Aug 14;15(16):3577. doi: 10.3390/nu15163577 (PMC10460006; doi:10.3390/nu15163577)
Supplement: Supplementary file 1 [file nutrients-15-03577-s001.zip › nutrients-2551310-supplementary.pdf]

Please find below the supplementary material of the article entitled 'Maternal bonding as a protective factor for orthorexia nervosa risk in dietetic students'.

Table S1. Association of ON risk as assessed by the ORTO-15 in Greek students of nutrition and dietetics (N=132) simultaneously with multiple variables (Multivariate logistic regression analysis).

Variable	Estimate	P value	OR (95% CI)
Gender [female]	-0,214	0,824	0.807 (0.121 - 5.366)
Age (years)	-0,084	0,354	0.92 (0.771 - 1.098)
BMI [normal weight]	2,449	0,044	11.581 (1.074 - 124.915)
BMI [overweight]	3,606	0,014	36.826 (2.059 - 658.506)
Years of Studies [>2]	0,462	0,56	1.586 (0.336 - 7.5)
Chronic Disease [yes]	-0,393	0,79	0.675 (0.038 - 12.074)
Medication [yes]	-0,526	0,628	0.591 (0.07 - 4.958)
Frequency Of Exercise [>4]	1,281	0,133	3.602 (0.677 - 19.173)
Duration Of Exercise [>1]	-0,717	0,253	0.488 (0.143 - 1.671)
STAIT [moderate anxiety]	2,373	0,002	10.727 (2.454 - 46.889)
STAIT [high anxiety]	1,699	0,014	5.467 (1.412 - 21.164)
MedDiet [sufficient adherence]	-0,262	0,707	0.77 (0.197 - 3.011)
EAT-26 [risk of eating disorders]	2,829	0,005	16.93 (2.397 - 119.577)
BI-AAQ-5 [risk of body image inflexibility]	1,51	0,034	4.527 (1.12 - 18.304)
Maternal Age (years)	0,037	0,657	1.038 (0.881 - 1.222)
Maternal BMI [overweight]	-0,233	0,714	0.792 (0.228 - 2.753)
Maternal BI-AAQ-5 [risk of body image inflexibility]	0,585	0,341	1.796 (0.538 - 5.999)
Maternal bonding (PBI) [affectionless control]	-1,023	0,294	0.36 (0.053 - 2.429)
Maternal bonding (PBI) [optimal parenting]	-1,391	0,134	0.249 (0.04 - 1.535)
Maternal bonding (PBI) [affectionate constraint]	-2,525	0,036	0.08 (0.008 - 0.852)
Paternal Age (years)	0,015	0,813	1.015 (0.899 - 1.146)
Paternal BMI [overweight]	1,18	0,115	3.254 (0.749 - 14.129)
Paternal BI-AAQ-5 [risk of body image inflexibility]	-0,437	0,573	0.646 (0.141 - 2.951)
Paternal bonding (PBI) [affectionless control]	0,917	0,246	2.502 (0.532 - 11.766)
Paternal bonding (PBI) [optimal parenting]	1,426	0,107	4.161 (0.736 - 23.523)
Paternal bonding (PBI) [affectionate constraint]	1,031	0,359	2.803 (0.31 - 25.339)

BMI=body mass index; MedDiet=Mediterranean diet; STAIT=State-Trait Anxiety Inventory; EAT-26=Eating Attitudes Test-26; BI-AAQ-5 = Body Image Acceptance & Action Questionnaire-5; PBI=Parental Bonding Instrument

* $p \leq 0.05$ was considered statistically significant.
